# Unusually swift response of relapsed Burkitt leukemia to R‐DHAP

**DOI:** 10.1002/jha2.501

**Published:** 2022-06-13

**Authors:** Dennis Christoph Harrer, Alexander Denk, Felix Keil, Karin Menhart, Stephanie Mayer, Daniel Wolff, Matthias Edinger, Wolfgang Herr, Matthias Grube

**Affiliations:** ^1^ Department of Medicine III‐Hematology and Oncology University Hospital Regensburg Regensburg Germany; ^2^ Department of Pathology University Hospital Regensburg Regensburg Germany; ^3^ Department of Nuclear Medicine University Hospital Regensburg Regensburg Germany

**Keywords:** allogeneic stem cell transplantation, Burkit leukemia, R‐DHAP, relapse, salvage therapy

## Abstract

Burkitt leukemia (BL) represents a highly aggressive lymphoma characterized by proliferation rates of around 100%, and a frequent spread into the central nervous system. If standard frontline chemotherapy fails, the prognosis is usually dismal, and reports on successful effective salvage therapy strategies for patients with relapsed/refractory BL are scant. Here, we report on a 40‐year‐old female patient who suffered an early relapse of BL three months after the completion of frontline chemoimmunotherapy. Strikingly, after only one cycle of R‐DHAP chemotherapy, the patient showed CR of BL enabling swift transition to a consolidating allogeneic stem cell transplantation. A 40‐year‐old previously healthy woman presented to the hospital with fatigue and incessant epistaxis, and a diagnosis of BL was made upon histological examination of a bone marrow biopsy. Treatment was initiated according to the GMALL 2002 B‐NHL/ALL protocol, which could induce complete molecular remission. Nevertheless, three months after chemotherapy, the patient exhibited BL relapse in the bone marrow, and on Fluorodeoxyglucose (FDG)–PET‐imaging. The relapse therapy was started with R‐DHAP, and after only one cycle, the patient once again entered complete remission (CR) paving the way for allogeneic stem cell transplantation. Unfortunately, the patient again relapsed five months after transplantation prompting salvage therapy with R‐DHAC and the execution of the second stem cell transplantation. However, one month after the second transplantation the patient presented with chemorefractory meningeosis leukemia resulting in the initiation of palliative care treatment. In summary, we report on rapid CR of relapsed BL after a single cycle of rituximab‐DHAP. Given a paucity of clinical trials on the treatment of patients with r/r BL, we intend to highlight the potential efficacy of rituximab‐DHAP as salvage therapy in those patients.

## 1 BACKGROUND

Burkitt leukemia/lymphoma (BL) represents an aggressive hematological malignancy associated with a dismal prognosis if frontline therapy fails [1, 2, 3, 4]. Standard BL therapy comprising a variety of different chemotherapeutic agents administered in combination with rituximab confers 5‐year overall survival (OS) rates of around 80% and complete remission (CR) rates over 80% [5, 6, 7]. In contrast, survival rates for patients with relapsed/refractory (r/r) BL are low with the only curative option being allogeneic stem cell transplantation (ASCT) executed after prior successful salvage therapy [2]. Nevertheless, reports on successful effective salvage therapy strategies for patients with r/r BL are scant. We report on a 40‐year‐old female patient who suffered an early relapse of BL three months after the completion of frontline chemoimmunotherapy. Salvage therapy was initiated with rituximab, dexamethasone, high dose cytarabine, and cisplatin (R‐DHAP). Strikingly, after only one cycle, the patient showed CR of BL enabling swift transition to the consolidating ASCT.

## 2 CASE PRESENTATION

**FIGURE 1:**
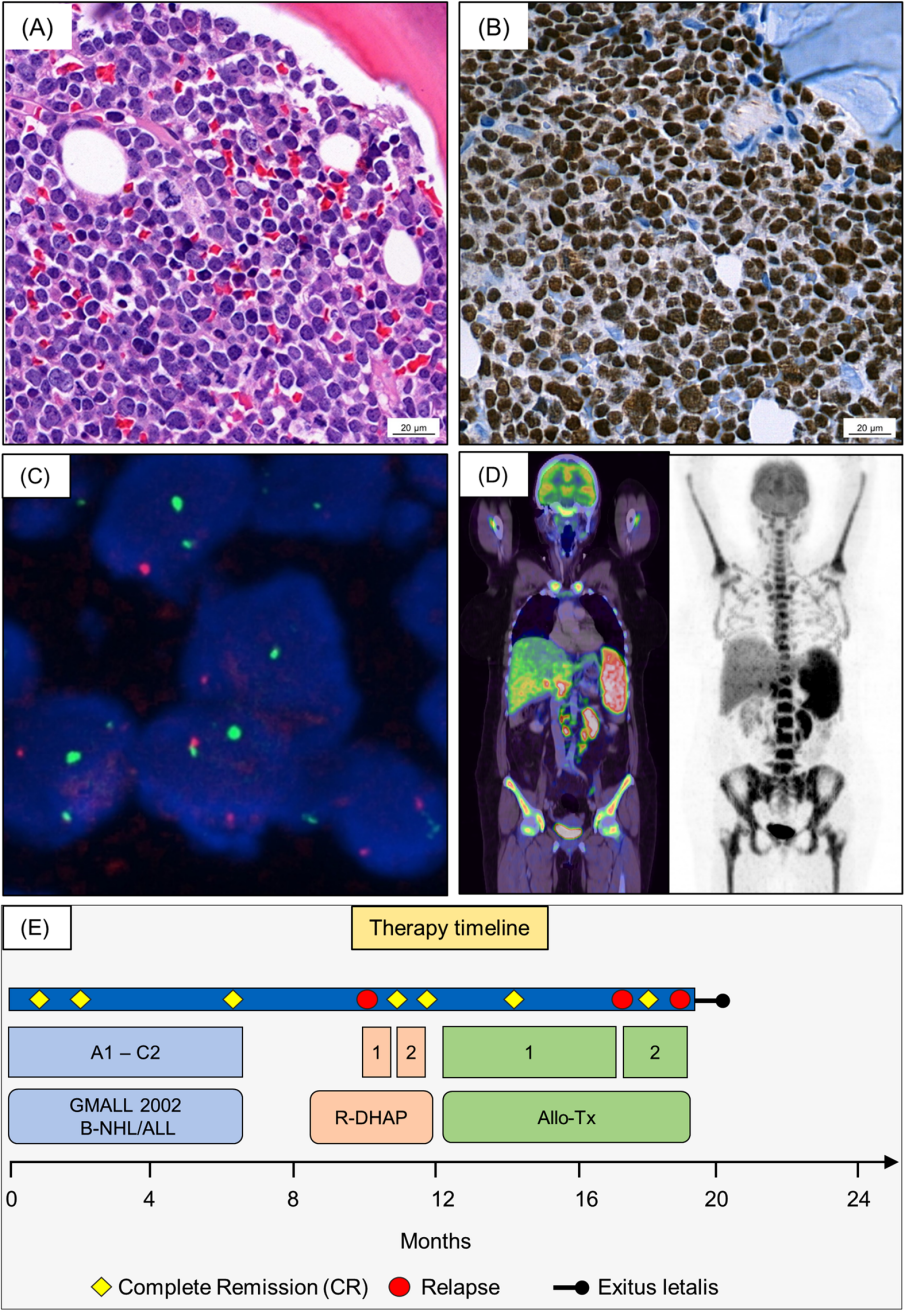
(A–C) Bone marrow biopsy at initial diagnosis. (A) H&E staining (400‐fold): subtotal bone marrow infiltration with a monomorphic population of medium‐sized mature lymphocytes showing basophilic cytoplasm, round nuclei with lacy chromatin and one or more small nucleoli. (B) Immunohistochemistry PAX5 (400‐fold): strong positivity for the B cell marker PAX5. (C) Multicolor break‐apart FISH: MYC rearrangement‐positive tumor cell, showing break‐apart signals. (D) FDG–PET‐imaging at initial diagnosis: multiple hypermetabolic lesions in lymph nodes on both sides of the diaphragm, in the spleen, in the bone marrow, and the liver. (E) Schematic therapy timeline depicting the different therapies and the corresponding treatment responses in the chronological order

**FIGURE 2:**
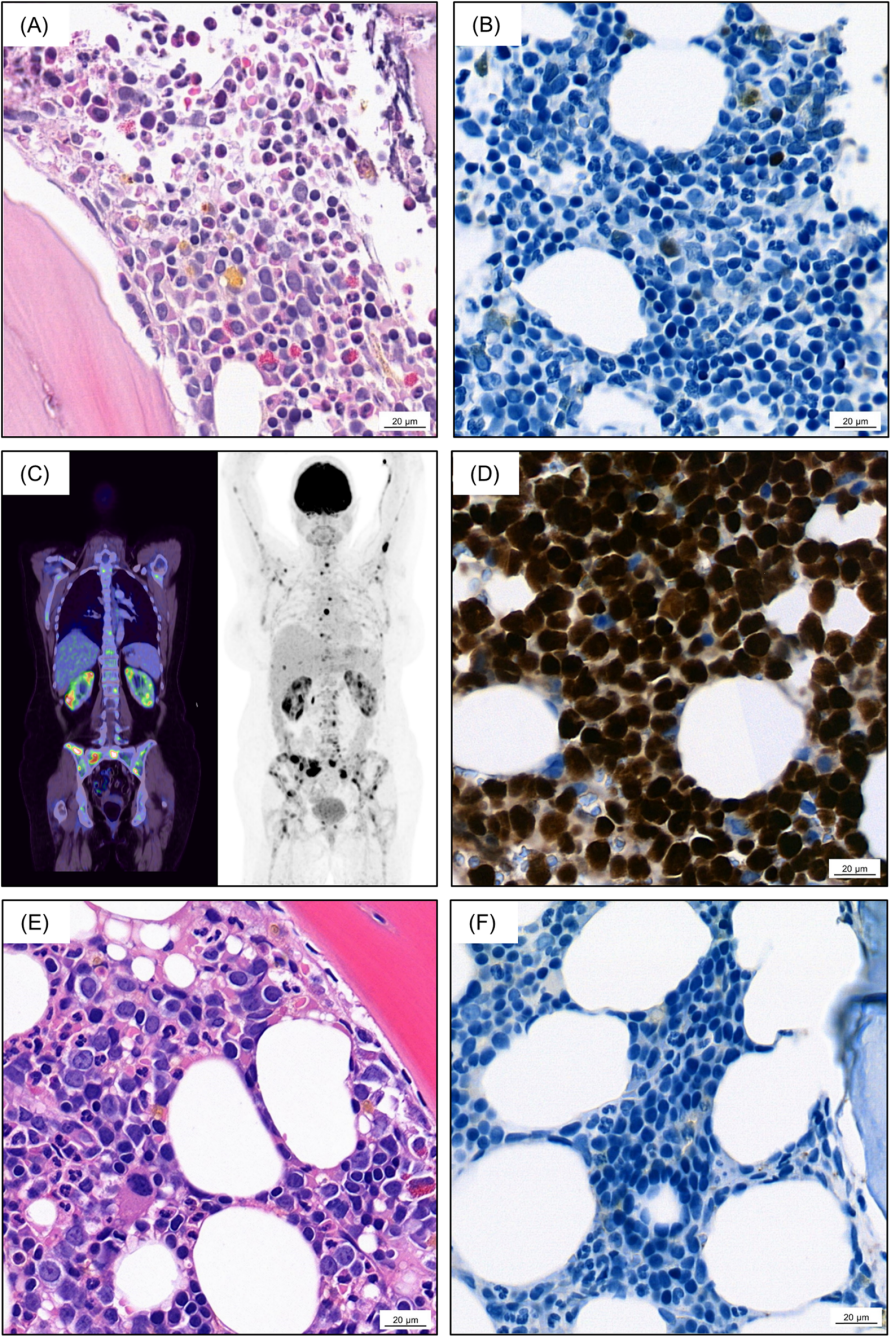
(A+B) Bone marrow biopsy after block A2 GMALL 2002 B‐NHL/ALL protocol. (A) H&E staining (400‐fold): regular bone marrow morphology indicating complete remission (CR). (B) Immunohistochemistry PAX5 (400‐fold): CR of pathological B cell infiltration. (C) FDG–PET‐imaging at initial relapse: metabolically active lesions in abdominal lymph nodes, in both kidneys and in the bone marrow. (D) Immunohistochemistry PAX5 (400‐fold): Burkitt leukemia relapse in the bone marrow. (E+F) Bone marrow biopsy after first cycle rituximab‐DHAP. (E) H&E staining (400‐fold): regular bone marrow morphology indicating CR. (F) Immunohistochemistry PAX5 (400‐fold): CR of pathological B cell infiltration

A 40‐year old previously healthy woman presented to the hospital with fatigue and incessant epistaxis in 2020. Physical examination revealed several hematomas. Thrombocytopenia, anemia, an elevated leukocyte count, and numerous large blast cells found in the peripheral blood raised the suspicion of acute leukemia. The subsequent bone marrow puncture showed an expansion (61%) of medium size and large vacuolated blast cells, which were characterized by flow cytometry as clonal (kappa light chain restriction), immature (CD45(+), HLA‐DR+, CD38+, CD10+, CD34−, TdT−) B cells (CD19+, CD20+, CD22(+), CD79a+, CD5−, cyIgM+). Histological examination of a bone marrow biopsy revealed a subtotal (> 95%) infiltration by an EBV‐negative high‐grade B cell lymphoma with a MYC‐rearrangement and an IGH‐BCL2 rearrangement (Figure 1A–C). Additionally, a 13q14 deletion and a TP53/17p deletion were found. FDG–Positron emission tomography (FDG–PET) showed multiple hypermetabolic lesions in lymph nodes on both sides of the diaphragm, in the spleen, in the bone marrow, and the liver (Figure 1D). No signs of central nervous system involvement emerged after Magnetic resonance imaging (MRI)‐imaging and cerebrospinal fluid examination. In aggregate, a diagnosis of mature B‐cell acute leukemia commonly referred to as BL was made in this patient. Therapeutically, chemotherapy according to the GMALL 2002 B‐NHL/ALL protocol was initiated (Figure 1E). First, a prephase therapy (cyclophosphamide, prednisone, and intrathecal triple therapy with methotrexate (MTX), cytarabine and dexamethasone) and block A1 (rituximab, dexamethasone, vincristine, ifosfamide, high‐dose MTX, cytarabine, etoposide, and a twice intrathecal triple therapy) were administered. Fortunately, after the first cycle of chemotherapy, a bone marrow aspiration showed complete cytological remission. Thus, chemotherapy was resumed with block B1 (rituximab, dexamethasone, vincristine, cyclophosphamide, high‐dose MTX, doxorubicin and a twice intrathecal triple therapy) and after another bone marrow analysis, which additionally confirmed molecular CR, with block C1 (rituximab, high‐dose MTX, vindesine, etoposide, cytarabine). Next, the patient received block A2 (equal to block A1), block B2 (equal to block B1), and block C2 (equal to block C1). Bone marrow analysis after block A2 (Figure 2A,B), and after six months of chemotherapy revealed ongoing CR. FDG–PET‐imaging showed complete regression of all metabolically active lesions. Three months after chemotherapy, the patient presented to the first follow‐up appointment without any subjective symptoms and with regular blood counts. However, FDG–PET‐imaging indicated BL relapse with metabolically active lesions in abdominal lymph nodes, both kidneys and the bone marrow (Figure 2C). Moreover, the histological examination of the additionally performed bone marrow biopsy once again showed subtotal infiltration with lymphatic blasts confirming the diagnosis of BL relapse just three months after completing extensive chemotherapy (Figure 2D). In this situation, the only curative option is conferred on by ASCT preceded by the remission‐inducing salvage therapy. On this account, the salvage therapy was attempted with rituximab‐DHAP (dexamethasone, high‐dose cytarabine, cisplatin; Figure 1E). Strikingly, a bone marrow biopsy obtained after the very first cycle rituximab‐DHAP showed CR (Figure 2E,F), and FDG–PET‐imaging showed clearance of all extramedullary lesions. Thus, the patient was prepared for ASCT while undergoing a second bridging cycle of rituximab‐DHAP. The final staging prior to ASCT confirmed the maintenance of CR. Afterward, matched unrelated donor ASCT could be performed. Molecular CR was confirmed two months after transplantation via bone marrow biopsy. Mild chronic skin GvHD arising during cyclosporine tapering was treated with prednisone. Five months after transplantation the patient was hospitalized for severe pain emanating from the bones. Flow cytometrical analysis of peripheral blood revealed BL relapse (blast percentage 6%). Based on the prior swift response to R‐DHAP, re‐induction chemotherapy was executed with R‐DHAC (exchange of cisplatin for carboplatin due to progressive renal insufficiency). Upon one cycle of R‐DHAC, the peripheral blood was cleared from BL blasts, and a second ASCT from the same donor was performed using cryopreserved stem cells. Acute skin GvHD grade two developed within a few weeks after the second ASCT and was treated with prednisolone. One month after the second ASCT, the patient was hospitalized for worsening general condition and acute kidney failure. Bone marrow biopsy once again revealed relapse of BL. Moreover, paralysis of the right leg prompted a diagnostic lumbar puncture, and analysis of cerebral spinal fluid revealed meningeosis leucemica. Despite immediate treatment with systemic dexamethasone and vindesine coupled with intrathecal injection of cytarabine, MTX and dexamethasone, the general condition as well as the vigilance of the patient deteriorated. Given the dismal prognosis, the treatment was switched to palliative care, and the patient died 20 months after receiving the diagnosis of BL.

## 3 DISCUSSION AND CONCLUSION

BL represents a highly aggressive lymphoma characterized by proliferation rates of around 100%, and a frequent spread into the central nervous system [3, 4, 8]. Hence, urgent initiation of chemotherapy is required. In Germany, therapy of BL is commonly guided by the B‐ALL/NHL 2002 clinical trial protocol developed by the German Multicenter Study Group on Adult Acute Lymphoblastic Leukemia (GMALL). Using this protocol, complete response rates (CR) of 88%, 5‐year OS rates of 80%, and progression‐free survival (PFS) rates of 71% can be achieved [5]. In case of relapsing or refractory BL, however, effective salvage strategies are missing, and few clinical data, which are mostly sourced from case reports and retrospective trials, are available to define the best treatment options in this situation.

In a retrospective analysis covering nine patients with r/r BL after treatment according to the GMALL B‐ALL/NHL 2002 trial protocol, Cremer et al. reported poor outcomes with no long‐term disease‐free survivors [2]. In total, eight patients were subjected to re‐induction chemotherapy, and only a single patient achieved a second CR allowing subsequent consolidation via allogeneic bone marrow transplantation. The remaining patients largely showed progressive disease despite salvage therapy, and rapidly succumbed to BL. The CR reported by Cremer et al. was induced upon the third cycle of R‐DHAP chemotherapy transitioning from a partial response (PR) in response to the first two R‐DHAP cycles. Precisely, four patients received platinum‐based intensive salvage therapy, only one responded achieving a delayed CR after the third cycle of rituximab‐DHAP. In the present case report, we show a striking CR after the very first cycle of rituximab‐DHAP without any significant chemotherapy‐associated toxicity in a patient with early relapse of BL. Based on their clinical observations, Cremer et al. argued that aggressive chemotherapy could not be the mainstay of BL salvage therapy discussing that palliative care might be the better approach for the majority of patients. Against the backdrop of the unusually swift response to R‐DHAP reported in the present case report, we believe that platinum‐based aggressive chemotherapy should not be dismissed as salvage therapy in patients with r/r BL. More importantly, achieving a second CR after relapse constitutes a crucial prerequisite for the success of a potential consolidating ASCT, which represents the only therapeutic option with curative potential in patients with r/r BL [9]. Therefore, the CR induced by R‐DHAP treatment paved the way for adopting a potentially curative approach in this young woman reported here. Unfortunately, the graft‐versus‐leukemia effect associated with ASCT was insufficient to prevent BL relapse in this patient. In another retrospective analysis evaluating 28 patients with r/r BL, the medium OS for patients with late (> 6 months) relapse amounted to 5.0 months, whereas patients with refractory disease/early relapse only had an OS of 1.4 months. Different salvage therapy regimes were employed including hyper‐CVAD (cyclophosphamide, vincristine, doxorubicin, dexamethasone), ICE (ifosfamide, carboplatin, etoposide), EPOCH (etoposide, prednisone, vincristine, cyclophosphamide, doxorubicin), and MOAD (MTX, vincristine, pegylated L‐asparaginase, dexamethasone) [1]. In this study, the overall response rate (ORR) of salvage chemotherapy in patients with late relapse and refractory disease/early relapse was 61% and 0%, respectively, underscoring the dire situation of patients with r/r BL.

In the context of this still maturing landscape of evidence, further case reports and clinical trials are urgently needed as the choice of salvage therapy regimens in patients with r/r BL still remains a highly individualized decision guided more by personal experience than by systematic evidence.

In summary, we report on rapid CR of relapsed BL after a single cycle of rituximab‐DHAP. Given a paucity of clinical trials on the treatment of patients with r/r BL, we intend to highlight the potential efficacy of rituximab‐DHAP as salvage therapy in those patients.

## CONFLICT OF INTEREST

The author declares that there is no conflict of interest that could be perceived as prejudicing the impartiality of the research reported.

## AUTHOR CONTRIBUTIONS

Dennis Christoph Harrer, Alexander Denk, Stephanie Mayer, Daniel Wolff, Matthias Edinger, Wolfgang Herr, and Matthias Grube treated the patient. Karin Menhart performed all imaging (nuclear medicine). Felix Keil analyzed all histopathological examinations. Dennis Christoph Harrer and Matthias Grube wrote the manuscript. All the authors revised the manuscript critically, approved the final manuscript, and agreed to be accountable for all aspects of the manuscript.

## ETHICS STATEMENT

Written informed consent was obtained from the patient prior to publication of this article.

## FUNDING

The authors received no specific funding for this work.

## PATIENT CONSENT

Informed consent for the publication of the case was obtained from the patient.

## Data Availability

The data that support the findings of this study are available from the corresponding author upon reasonable request.
